# Supplementary material for: Examining the effectiveness of the Gateway conditional caution on health and well-being of young adults committing low-level offences: a randomised controlled trial
Source: BMJ Open. 2024 Apr 25;14(4):e081179. doi: 10.1136/bmjopen-2023-081179 (PMC11057255; doi:10.1136/bmjopen-2023-081179)
Supplement: Supplementary data [file bmjopen-2023-081179supp001.pdf]

Appendix A: Delivery of Gateway and usual process

Table 1: Conditions attached to cautions, presented by whether the participant received a Gateway conditional caution or a caution forming part of usual process (either a simple caution or a different conditional caution).

	**Gateway conditional caution (n=105)**	**Usual process (n=80)**
**Conditions attached (multiple conditions possible), n (%)**		
Standard Gateway conditions (no additional conditions added)	85 (81.0)	NA
None (simple caution)	NA	5 (6.3)
Compensation	18 (17.1)	20 (25.0)
Letter of apology	5 (4.8)	10 (12.5)
Victim awareness course	0 (0)	14 (17.5)
Alcohol diversion course	0 (0)	11 (13.8)
Drugs diversion course	0 (0)	16 (20.0)
Not to enter specific premises	0 (0)	1 (1.3)
Fine	0 (0)	5 (6.3)
Women and Desistance Empowerment programme	0 (0)	9 (11.3)
Restorative justice	0 (0)	0 (0)

Table 2: Information on delivery of the Gateway intervention.

	**Received Gateway conditional caution (n=105)**
**LINX workshops attended (supplemented with change of status data)**	
**Number with data, n (%)**	**101 (96.2)**
0 (Did not attend LINX sessions due to COVID-19 pause)	4 (4.0)
0 (participant chose to not attend LINX sessions)	8 (7.9)
1 (participant chose not to attend LINX session)	1 (1.0)
2	88 (87.1)
**Delivery of LINX workshops**	
**Number with data, n (% of those who attended at least one workshop)**	**80 (89.9%)**

Face-to-face	45 (56.3)
Telephone	35 (43.8)
**Contacts attempted by navigator (excluding LINX workshops)**	
**Number with data, n (%)**	**76 (72.4)**
Mean (SD)	52.8 (25.0)
Median (IQR)	42 (39, 63)
Min, Max	22, 168
**Successful contacts made by navigator (excluding LINX workshops)**	
**Number with data, n (%)**	**76 (72.4)**
Mean (SD)	26.0 (20.7)
Median (IQR)	19 (15, 31)
Min, Max	0, 108
**Total duration of successful contacts, minutes**	
Number with data, n (%)	**70 (66.7)**
Mean (SD)	761.5 (594.6)
Median (IQR)	626.5 (380, 978)
Min, Max	36, 2785

Appendix B: Participants informed of their disposal decision after their 4-week follow-up was due

Table 3: Information on time between randomisation and disposal decision and whether the 4-week follow-up was attended, for those informed of their disposal decision after the 4-week follow-up was due.

	**Gateway conditional caution (n=12)**	**Usual process (n=3)**	**Total (n=15)**
**Time between randomisation and disposal, days**			
**Number with data (%)**	**12 (100)**	**3 (100)**	**15 (100)**
Mean (SD)	49.6 (18.1)	NA	NA
Median (IQR)	42 (34.5, 67.5)	NA	NA
Min, Max	29, 77	NA	NA
**Attended 4-week follow-up, n (%)**			
**Number with data (%)**	**12 (100)**	**3 (100)**	**15 (100)**

Yes	8 (66.7)	NA	NA
No	4 (33.3)	NA	NA

Appendix C: Index of Multiple Drug Use

Table 4: Index of Multiple Drug Use presented at 4-weeks, 16-weeks and 1-year post randomisation.

	**Gateway conditional caution (n=109)**	**Usual process (n=82)**
**Week 4**		
**Number with data, n (%)**	**57 (52.3)**	**36 (43.9)**
Mean (SD)	23.3 (6.4)	21.3 (5.0)
Median (IQR)	22 (18, 27)	21.5 (16.5, 25)
Min, Max	15, 42	15, 31
**Week 16**		
**Number with data, n (%)**	**54 (49.5)**	**39 (47.6)**
Mean (SD)	23.3 (7.5)	22.3 (5.9)
Median (IQR)	21 (17, 27)	22 (16, 25)
Min, Max	15, 47	15, 38
**Year 1**		
**Number with data, n (%)**	**27 (24.8)**	**16 (19.5)**
Mean (SD)	25.2 (7.7)	25.8 (6.3)
Median (IQR)	23 (18, 31)	25.5 (21, 28.5)
Min, Max	16, 41	16, 38

Appendix D: Adverse childhood experiences

Table 5: Adverse childhood experiences reported at 16 weeks post-randomisation.

	**Gateway conditional caution (n=109)**	**Usual process (n=82)**
**Number of adverse childhood experiences**		
**Number with data (%)**	**54 (49.5)**	**39 (47.6)**
Mean (SD)	3.0 (2.6)	3.6 (3.0)
Median (IQR)	2 (1, 5)	4 (1, 5)
Min, Max	0, 10	0, 11

Appendix E: Health economic analysis

Table 6: Health economic data at 4-weeks, 16-weeks and 1-year post-randomisation, presented by group.

	**4-weeks post-randomisation**	**16-weeks post-randomisation**	**1-year post-randomisation**

	**Gateway conditional caution (n=109)**	**Usual process (n=82)**	**Gateway conditional caution (n=109)**	**Usual process (n=82)**	**Gateway conditional caution (n=109)**	**Usual process (n=82)**
**Employed in previous month**						
**Number with data, n (%)**	**57 (52.3)**	**36 (43.9)**	**54 (49.5)**	**39 (47.6)**	**27 (24.8)**	**16 (19.5)**
Yes	31 (54.4)	16 (44.4)	31 (57.4)	19 (48.7)	16 (59.3)	11 (68.8)
No	26 (45.6)	20 (55.6)	23 (42.6)	20 (51.3)	11 (40.7)	5 (31.3)
**Number of times visited GP in previous month**						
**Number with data, n (%)**	**57 (52.3)**	**36 (43.9)**	**53 (48.6)**	**39 (47.6)**	**27 (24.8)**	**15 (18.3)**
Mean (SD)	0.4 (0.7)	0.5 (1.0)	0.4 (1.0)	0.5 (0.9)	0.5 (1.0)	1.3 (2.6)
Median (IQR)	0 (0, 1)	0 (0, 0.5)	0 (0, 0)	0 (0, 0)	0 (0, 1)	1 (0, 1)
Min, Max	0, 3	0, 4	0, 5	0, 3	0, 4	0, 10
**Number of times used drug/alcohol services in previous month**						
**Number with data, n (%)**	**56 (51.4)**	**36 (43.9)**	**53 (48.6)**	**39 (47.6)**	**26 (23.9)**	**15 (18.3)**
Mean (SD)	0.3 (0.9)	0.3 (1.7)	0.4 (1.2)	0.1 (0.4)	0.2 (0.8)	0.4 (1.1)
Median (IQR)	0 (0, 0)	0 (0, 0)	0 (0, 0)	0 (0, 0)	0 (0, 0)	0 (0, 0)
Min, Max	0, 4	0, 10	0, 5	0, 2	0, 4	0, 4
**Number of times visited accident and emergency in previous month**						
**Number with data, n (%)**	**57 (52.3)**	**36 (43.9)**	**54 (49.5)**	**39 (47.6)**	**27 (24.8)**	**15 (18.3)**
Mean (SD)	0.2 (0.9)	0.1 (0.2)	0.1 (0.3)	0 (0.2)	0.6 (1.9)	0.2 (0.6)
Median (IQR)	0 (0, 0)	0 (0, 0)	0 (0, 0)	0 (0, 0)	0 (0, 0)	0 (0, 0)
Min, Max	0, 6	0, 1	0, 2	0, 1	0, 10	0, 2
**Number of times admitted to hospital as inpatient in previous month**						
**Number with data, n (%)**	**57 (52.3)**	**36 (43.9)**	**53 (48.6)**	**39 (47.6)**	**27 (24.8)**	**15 (18.3)**
Mean (SD)	0.1 (0.3)	0 (0)	0.1 (0.3)	0 (0)	0.3 (1.0)	0 (0)
Median (IQR)	0 (0, 0)	0 (0, 0)	0 (0, 0)	0 (0, 0)	0 (0, 0)	0 (0, 0)
Min, Max	0, 2	0, 0	0, 2	0, 0	0, 4	0, 0
**Number of times used community mental health**						

**team in previous month**						
**Number with data, n (%)**	**56 (51.4)**	**35 (2.7)**	**53 (48.6)**	**38 (46.3)**	**26 (23.9)**	**15 (18.3)**
Mean (SD)	0.2 (0.8)	0.2 (0.7)	0.2 (0.6)	1.1 (4.9)	0.4 (1.1)	0.5 (1.2)
Median (IQR)	0 (0, 0)	0 (0, 0)	0 (0, 0)	0 (0, 0)	0 (0, 0)	0 (0, 0)
Min, Max	0, 4	0, 3	0, 3	0, 30	0, 4	0, 4
**Number of times used psychiatric services as in-patient in previous month**						
**Number with data, n (%)**	**57 (52.3)**	**36 (43.9)**	**53 (48.6)**	**39 (47.6)**	**27 (24.8)**	**15 (18.3)**
Mean (SD)	0 (0.2)	0 (0.2)	0 (0)	0.2 (1.0))	0 (0.2)	0.1 (0.3)
Median (IQR)	0 (0, 0)	0 (0, 0)	0 (0, 0)	0 (0, 0)	0 (0, 0)	0 (0, 0)
Min, Max	0, 1	0, 1	0, 0	0, 6	0, 1	0, 1
**Used the following prescribed medications in previous month, n (%)**						
**Number with data, n (%)**	**57 (52.3)**	**36 (43.9)**	**54 (49.5)**	**39 (47.6)**	**27 (25.0)**	**16 (19.3)**
Amitriptyline	1 (1.8)	0 (0)	1 (1.9)	0 (0)	2 (7.4)	0 (0)
Aripirazole	1 (1.8)	0 (0)	0 (0)	0 (0)	0 (0)	0 (0)
Cerelle	0 (0)	0 (0)	0 (0)	0 (0)	1 (3.7)	0 (0)
Citalopram	3 (5.3)	1 (2.8)	1 (1.9)	2 (5.1)	1 (3.7)	0 (0)
Co-codamol	0 (0)	0 (0)	1 (1.9)	0 (0)	0 (0)	0 (0)
Codeine	0 (0)	1 (2.8)	0 (0)	0 (0)	0 (0)	0 (0)
Cyclizine	1 (1.8)	0 (0)	0 (0)	0 (0)	0 (0)	0 (0)
Diazepam	0 (0)	0 (0)	0 (0)	1 (2.6)	0 (0)	0 (0)
Doxycycline	0 (0)	0 (0)	0 (0)	1 (2.6)	0 (0)	0 (0)
Inhaler	0 (0)	4 (11.1)	5 (9.3)	2 (5.1)	1 (3.7)	0 (0)
Escitalopram	1 (1.8)	1 (2.8)	0 (0)	0 (0)	0 (0)	0 (0)
Fluoxetine	3 (5.3)	1 (2.8)	0 (0)	2 (5.1)	0 (0)	0 (0)
Quetiapine	2 (3.5)	1 (2.8)	0 (0)	0 (0)	0 (0)	1 (6.3)
Lamotrigine	0 (0)	0 (0)	0 (0)	0 (0)	1 (3.7)	0 (0)
Lymecycline	0 (0)	2 (5.6)	0 (0)	1 (2.6)	0 (0)	0 (0)
Macrogol 3350	1 (1.8)	0 (0)	0 (0)	0 (0)	0 (0)	0 (0)
Melatonin	0 (0)	0 (0)	0 (0)	1 (2.6)	0 (0)	0 (0)
Methadone	0 (0)	0 (0)	1 (1.9)	0 (0)	0 (0)	0 (0)
Mirtazapine	2 (3.5)	0 (0)	2 (3.7)	0 (0)	1 (3.7)	1 (6.3)
Naproxen	1 (1.8)	0 (0)	2 (3.7)	0 (0)	0 (0)	0 (0)
Omeprazole	1 (1.8)	0 (0)	0 (0)	0 (0)	0 (0)	0 (0)
Ondansetron	0 (0)	0 (0)	1 (1.9)	0 (0)	0 (0)	0 (0)
Olanzapine	0 (0)	1 (2.8)	0 (0)	0 (0)	0 (0)	0 (0)
Phenergan	0 (0)	2 (5.6)	0 (0)	0 (0)	0 (0)	0 (0)

Prednisolone	1 (1.8)	0 (0)	0 (0)	0 (0)	0 (0)	0 (0)
Pregabalin	0 (0)	1 (2.8)	1 (1.9)	0 (0)	0 (0)	0 (0)
Prochlorperazine maleate	1 (1.8)	0 (0)	0 (0)	0 (0)	0 (0)	0 (0)
Promethazine hydrochloride	0 (0)	0 (0)	0 (0)	1 (2.6)	0 (0)	0 (0)
Propranolol hydrochloride	1 (1.8)	0 (0)	0 (0)	0 (0)	0 (0)	0 (0)
Quetiapine	2 (3.5)	0 (0)	4 (7.4)	3 (7.7)	2 (7.4)	0 (0)
Ramipril	0 (0)	0 (0)	1 (1.9)	0 (0)	0 (0)	0 (0)
Risperidone	0 (0)	1 (2.8)	0 (0)	0 (0)	0 (0)	0 (0)
Salbutamol	0 (0)	0 (0)	0 (0)	0 (0)	0 (0)	1 (6.3)
Sertraline	3 (5.3)	4 (11.1)	7 (13.0)	5 (12.8)	2 (7.4)	2 (12.5)
Prochlorperazine	0 (0)	0 (0)	1 (1.9)	0 (0)	0 (0)	0 (0)
Tacrolimus	1 (1.8)	0 (0)	0 (0)	0 (0)	0 (0)	0 (0)
Venlafaxine	1 (1.8)	0 (0)	0 (0)	1 (2.6)	1 (3.7)	0 (0)
Vortioxetine	0 (0)	1 (2.8)	0 (0)	0 (0)	0 (0)	0 (0)
**Reason for using prescribed medications in previous month, n (%)**						
**Number with data (% of those who reported using a medication)**						
Acne	0 (0)	3 (20.0)	0 (0)	0 (0)	0 (0)	0 (0)
Anterior cruciate ligament injury	0 (0)	1 (6.7)	0 (0)	0 (0)	0 (0)	0 (0)
ADHD	1 (5.0)	1 (6.7)	1 (4.8)	0 (0)	0 (0)	0 (0)
Anxiety	7 (35.0)	7 (46.7)	4 (19.0)	2 (14.3)	2 (25.0)	2 (28.6)
Asthma	1 (5.0)	4 (26.7)	5 (23.8)	2 (14.3)	1 (12.5)	1 (14.3)
Back pain	0 (0)	0 (0)	1 (4.8)	0 (0)	0 (0)	0 (0)
Blood pressure	0 (0)	0 (0)	0 (0)	0 (0)	1 (12.5)	0 (0)
Depression	11 (55.0)	7 (46.7)	8 (38.1)	3 (21.4)	5 (62.5)	2 (28.6)
Ear infection	0 (0)	0 (0)	0 (0)	0 (0)	0 (0)	1 (14.3)
Gastroparesis	1 (5.0)	0 (0)	1 (4.8)	0 (0)	1 (12.5)	0 (0)
Heroin addiction	0 (0)	0 (0)	1 (4.8)	0 (0)	0 (0)	0 (0)
Hypertension	0 (0)	0 (0)	1 (4.8)	0 (0)	0 (0)	0 (0)
Immune	1 (5.0)	0 (0)	1 (4.8)	0 (0)	0 (0)	0 (0)

system suppression post-kidney transplant						
Inflammation	1 (5.0)	0 (0)	0 (0)	0 (0)	0 (0)	0 (0)
Insomnia	2 (10.0)	1 (6.7)	0 (0)	1 (7.1)	1 (12.5)	0 (0)
Mood stabilisation	2 (10.0)	1 (6.7)	3 (14.3)	1 (7.1)	1 (12.5)	0 (0)
Nail infection	0 (0)	0 (0)	0 (0)	0 (0)	0 (0)	1 (14.3)
Nausea	1 (5.0)	0 (0)	1 (4.8)	0 (0)	0 (0)	0 (0)
Pain relief	0 (0)	0 (0)	2 (9.5)	0 (0)	1 (12.5)	0 (0)
Panic attacks	0 (0)	1 (6.7)	0 (0)	0 (0)	0 (0)	0 (0)
Psychosis	2 (10.0)	1 (6.7)	1 (4.8)	0 (0)	1 (12.5)	1 (14.3)
PTSD	0 (0)	2 (13.3)	0 (0)	0 (0)	0 (0)	0 (0)
